# Identifying a list of *Salmonella* serotypes of concern to target for reducing risk of salmonellosis

**DOI:** 10.3389/fmicb.2024.1307563

**Published:** 2024-02-12

**Authors:** Tatum S. Katz, Dayna M. Harhay, John W. Schmidt, Tommy L. Wheeler

**Affiliations:** U.S. Department of Agriculture, Agricultural Research Service, Roman L. Hruska U.S. Meat Animal Research Center, Clay Center, NE, United States

**Keywords:** non-typhoidal salmonellosis, *Salmonella enterica*, serotypes, machine learning, public health, epidemiology

## Abstract

There is an increasing awareness in the field of *Salmonella* epidemiology that focusing control efforts on those serotypes which cause severe human health outcomes, as opposed to broadly targeting all *Salmonella*, will likely lead to the greatest advances in decreasing the incidence of salmonellosis. Yet, little guidance exists to support validated, scientific selection of target serotypes. The goal of this perspective is to develop an approach to identifying serotypes of greater concern and present a case study using meat- and poultry-attributed outbreaks to examine challenges in developing a standardized framework for defining target serotypes.

## 1 Introduction

In the United States, non-typhoidal *Salmonella* (NTS) is the leading cause of bacterial foodborne illness (Centers for Disease Control and Prevention, 2023b) with $4.1 billion lost to NTS illness yearly (United States Department of Agriculture Economic Research Service, 2023). Despite numerous improvements in the control of *Salmonella* cross-contamination in food processing and production environments, NTS illness rates have not decreased in the last 20 years (Centers for Disease Control and Prevention, 2023c). This indicates that scientific understanding of NTS throughout affected food production and processing systems has not reached the level that enables effective control strategies.

A complicating factor in *Salmonella* control is the diversity of the genus. Consisting of two species and seven subspecies, *Salmonella* are further subtyped by serotyping, a phenotyping method which determines agglutination of the bacteria with antisera to identify antigenic variants (Grimont and Weill, 2007). Less than 2% of the >2,600 known serotypes consistently appear in reports on U.S. human infections (Issenhuth-Jeanjean et al., 2014; Centers for Disease Control and Prevention, 2023d). New information on genomic differences among *Salmonella* serotypes (den Bakker et al., 2011; Suez et al., 2013; Cheng et al., 2019; Rakov et al., 2019; Wang et al., 2020) has led to specifically targeting *Salmonella* that pose the greatest risk to human health, rather than broadly managing all *Salmonella* contamination. To this end, industry, academia, and government organizations have begun to focus research efforts on determining which serotypes to target and methods for rapidly identifying those serotypes of greater concern (Cohn et al., 2021; Chen et al., 2022; United States Department of Agriculture Food Safety and Inspection Service, 2022; Centers for Disease Control and Prevention, 2023a).

Analysis of epidemiological data may identify *Salmonella* serotypes with a greater impact on human health. A key original epidemiological analysis determined that there were significant differences among serotypes in their epidemiological outcomes by analysis of 1996 to 2006 FoodNet data (Jones et al., 2008). The Jones study presented a unique and practical approach based on retrospective, epidemiological data, but was limited to data collected from 10 states (representing 10–15% of the US population) over 11 years. Furthermore, FoodNet data consists of sporadic illnesses which are not necessarily part of an identified outbreak, and are not confirmed to be transmitted by food (Centers for Disease Control and Prevention, 2021). Given the evolving *Salmonella* regulatory landscape and the limitations of the Jones study, we have revisited this epidemiological analysis using new data and with a new goal: identification of serotypes to target for management to improve human health outcomes (which we will refer to throughout as “serotypes of concern” or SoC). In our analyses, we focus on salmonellosis outbreaks across the United States with a confirmed food transmission route utilizing the CDC's National Outbreak Reporting System (Centers for Disease Control and Prevention, 2023d). Accordingly, we present two different methods for analyzing CDC *Salmonella* outbreak data collected between 2009–2021 and attributed to meat and poultry (8,524 illnesses across 36 serotypes). During these analyses, we also identified several obstacles that complicate the conclusions made. The goals of this perspective, therefore, are to (1) suggest serotypes of concern associated with meat and poultry; (2) outline some of the obstacles and opportunities in determining SoC using epidemiological data.

## 2 Statistical approaches using epidemiological data

There is no consensus on what constitutes a SoC. The Jones study, while providing a powerful framework for examining differences across serotypes by epidemiological data, did not produce a definitive list of target serotypes (Jones et al., 2008). The USDA FSIS has identified their most commonly-detected serotypes Infantis, Enteritidis, and Typhimurium as Key Performance Indicators (KPIs), yet these together represented just 4.22% of *Salmonella* positive FSIS samples from 2020 to 2021, and 26% of all-cause sporadic illness isolates in 2020 (United States Department of Agriculture Food Safety and Inspection Service, 2022; Centers for Disease Control and Prevention, 2023a). Furthermore, much of the scientific literature on *Salmonella* virulence and host-pathogen interaction focuses on two of the highest-incidence serotypes (Typhimurium and Enteritidis), with little study devoted to other serotypes that contribute to most human illnesses in the US each year.

To provide an actionable list of SoC, quantitative validation is key. We define two major challenges to achieving this list: first, the list should be complete enough that targeting the serotypes included would result in decreasing salmonellosis to meet the DHHS Healthy People 2030 goals (U. S. Department of Health and Human Services, 2020) but concise enough to be actionable; and second, the determination of which epidemiological variables are critical for reducing outbreaks and illnesses. We have developed two methods for identifying SoC: a machine learning-based approach and a more classical, outlier-based approach. These approaches represent two extremes of statistical approaches to this problem: the machine learning approach is highly flexible and less constrained by researcher input, while the outlier approach brings together researcher input and quantitative validation in a simple decision rule. The results of both approaches were combined to create a priority SoC list that could be targeted for reducing U.S. illnesses attributed to chicken, turkey, pork, and beef.

### 2.1 Epidemiological data

Data representing all 50 U.S. states and Puerto Rico were downloaded from Centers for Disease Control's National Outbreak Reporting System (NORS) on 1/18/2023 (Centers for Disease Control and Prevention, 2023d). This dataset contained information on the confirmed or suspected food source, categorized following the Integrated Food Safety Analytics Collaboration (IFSAC) Food Categorization Scheme (Richardson et al., 2017), as well as the year of the first illness, the confirmed or suspected *Salmonella* serotype(s) responsible, and information on the outbreak including the number ill, number hospitalized, and number of deaths. We focus on outbreak-associated cases because we seek to identify those serotypes which cause systematic illness (i.e., we can examine source attribution as a variable) compared to serotypes which cause sporadic cases, where the source is generally unknown. Following data cleaning, including removal of observations with missing epidemiological, attribution, or etiology data, 694 out of 3042 outbreaks remained for analysis across all attributed sources (Supplementary material).

### 2.2 Machine learning approach

Detailed methods for both approaches are available in the Supplementary material, and all data and code generated for this study is available for free download at https://github.com/tatumskatz/serotypesOfConcern. Machine learning methods are especially useful for uncovering previously unnoticed trends and patterns in complex biological data since minimal assumptions have to be made about the data generation process (Bzdok et al., 2018). In this approach, we utilized an agglomerative nesting hierarchical cluster analysis (AGNES) to categorize serotypes as SoC or not (Supplementary material). Hierarchical cluster analysis proceeds by calculating the similarity between observations over multiple variables, and then using those similarity scores to group observations. Each observation starts off alone and is iteratively grouped with others based on distance (Altman and Krzywinski, 2017). After groups are created, the optimal number of groups is determined by assessing how similar the observations in a group are to each other and by maximizing within-group similarity while minimizing between-group similarity (Altman and Krzywinski, 2017). Once the optimal number of groups was determined, we then used expert knowledge to identify the group which contains the SoC so that any serotype in that group is classified as a SoC. By using a flexible, pattern-seeking approach and allowing the researcher to only provide input at the very end, this method can potentially reveal new insights in this complex dataset.

#### 2.2.1 Machine learning results

**Figure 1:**
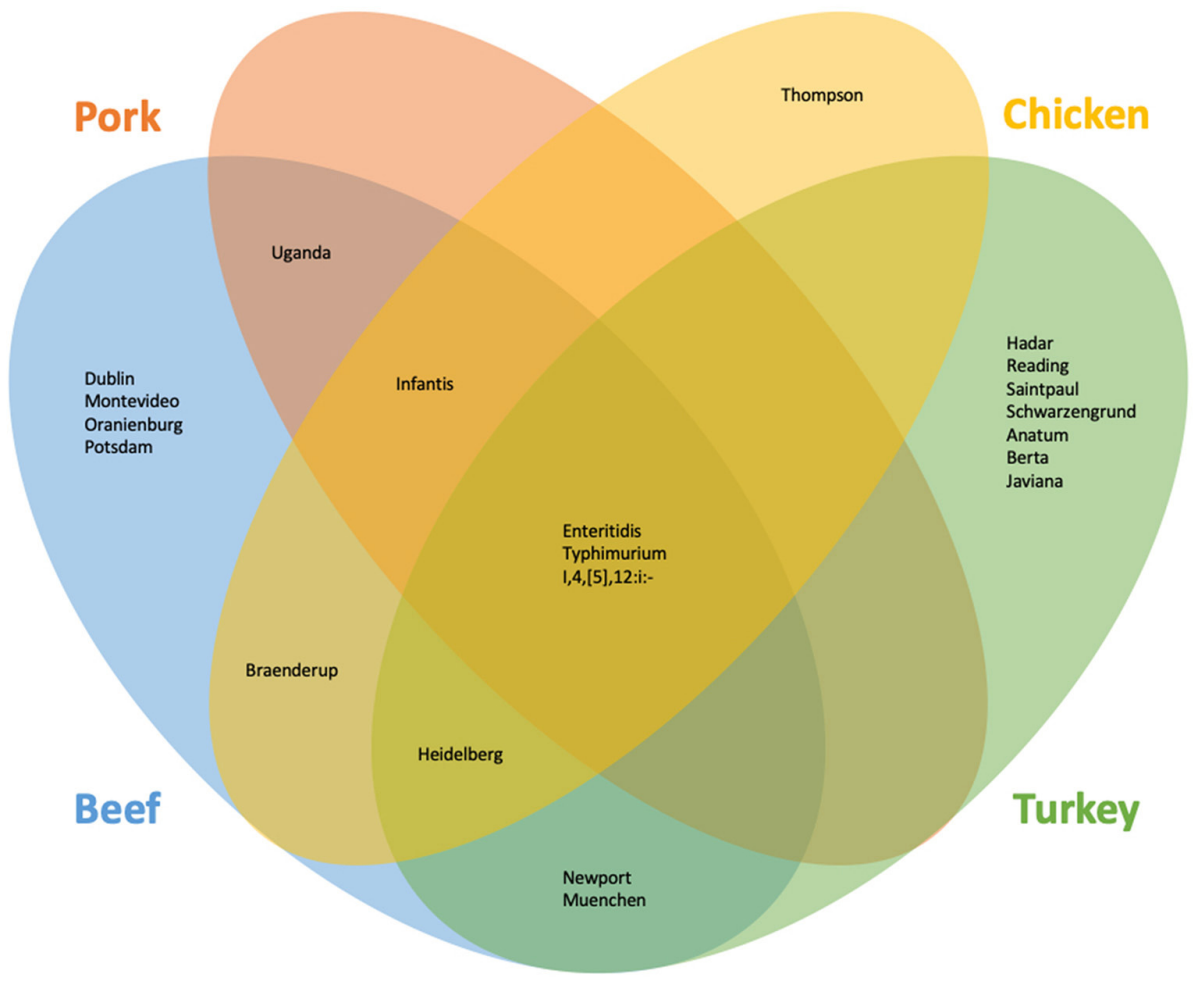
Serotypes identified as of concern using the machine learning and outlier approach for beef, chicken, pork, and turkey.

SoC were identified for meat overall and each commodity. In all cases, the Ward cluster method outperformed other methods and so was used to generate all clusters. Optimal number of groups ranged from 3 for meat overall, beef, chicken, and turkey to 4 for pork. Decision rules for categorizing serotypes as SoC varied by commodity: for meat overall, beef, chicken, and turkey “more than one outbreak in at least 2 years”; and for pork, “more than one outbreak in at least 2 years, or one outbreak with at least 7 hospitalizations in at least 2 years”. The SoC lists also varied by commodity: SoC for the four commodities and meat overall are presented in Table 1. Notably, the list for chicken SoC included only Enteritidis (Supplementary Table 1). Further analysis showed that the large number of Enteritidis outbreaks attributable to chicken products appeared to overwhelm other outbreaks so that no other serotypes are identified. Conversely, commodities with fewer outbreaks overall, and beef especially, revealed unanticipated SoC including Dublin and Uganda. Comparisons of the epidemiological data of these serotypes with established outbreak serotypes such as Enteritidis and Typhimurium revealed similar values for outbreaks, illnesses, and hospitalizations within the beef commodity (Table 1, Figure 1).

**Table 1:** Summary statistics for identified serotypes of concern attributed to meat and poultry from the CDC NORS dataset.

**Serotype**	**Number of outbreaks**	**Number of illnesses**	**Number of hospitalizations**	**Hospitalization to illness ratio**	**Proportion of total illnesses**	**Identifying approach**	**Commodity (ML approach)**	**Commodity (outlier approach)**
**Enteritidis**	65	1,848	204	0.11	0.22	Both	Meat overall, beef, chicken, pork, turkey	Beef, turkey
**Typhimurium**	24	864	127	0.15	0.10	Both	Meat overall, beef, pork, turkey	Beef, chicken, turkey
**I,4,[5],12:i:-**	19	751	138	0.18	0.09	Both	Meat overall, pork, turkey	Beef, chicken, pork
**Heidelberg**	17	1,380	386	0.27	0.17	Both	Meat overall, turkey	Beef, chicken, turkey
**Infantis**	9	334	62	0.19	0.04	Both	Meat overall	Beef, chicken, pork
**Newport**	13	856	224	0.26	0.10	Both	Meat overall, beef	Beef, turkey
Uganda	5	67	11	0.16	0.01	Both	Beef	Beef
**Braenderup**	8	133	23	0.17	0.02	Outlier		Beef, chicken
**Muenchen**	4	119	6	0.05	0.01	Outlier		Beef, turkey
Montevideo^*^	4	441	3	0.01	0.05	Both	Meat overall	Beef, chicken
Javiana	5	113	46	0.41	0.01	Both	Meat overall	Chicken, turkey
**Reading**	3	375	133	0.35	0.05	Both	Turkey	Meat overall, turkey
**Dublin**	2	51	16	0.31	0.01	Both	Beef	Beef
Oranienburg	1	18	2	0.11	0.00	Outlier		Beef
Potsdam	1	9	1	0.11	0.00	Outlier		Beef
Thompson	5	166	19	0.11	0.02	Outlier		Chicken
Saintpaul	3	83	16	0.19	0.01	Both	Turkey	Chicken, turkey
**Hadar**	2	51	12	0.24	0.01	Both	Turkey	Turkey
Schwarzengrund	3	53	0	0.00	0.01	Outier		Pork, turkey
Anatum	2	12	1	0.08	0.00	Outlier		Turkey
Berta	2	65	8	0.12	0.01	Outlier		Pork, turkey
Total	197	7,789	1,216		0.95			

^*^See additional information in *Data Limitations*. Bolded serotypes were identified by CDC BEAM as moderate to high illness burden SoC for one or more of the four commodities on 08/29/2023.

### 2.3 Outlier approach

If the machine learning approach produces a “black box” decision rule, the outlier approach produces a “clear box” decision rule. Outliers have no single mathematical definition, but we can intuitively describe them as observations which are so different from the rest of the data that they seem to be generated by an entirely different process (Hawkins, 1980). To define an outlier, we must have an idea of what “normal” observations are; then, we can identify abnormal observations (Aggarwal, 2017). By allowing for an *a priori* model of a “normal” data generation process (i.e., what a “normal” outbreak looks like), we can incorporate expert knowledge into our SoC definition and still allow quantitative validation by generating a score for how outlied each “abnormal” serotype is. Outlier identification is challenging for a variety of reasons, but we meet a specific challenge for our serotype list: many outlier tests assume only one or two outliers exist (Aggarwal, 2017); yet, we do not want to pre-define the number of outliers. Therefore, we utilized an approach which defines outliers using quartiles. This method avoids specifying a distribution (which is challenging given the nature of the data) or number of outliers and instead calculates how different each observation is from the rest of the data. For our case study, we used the average outbreak size and hospitalization:illness ratio to determine target serotypes. This method is similar, and sometimes identical to, the way one might use a box-and-whisker plot to identify outliers (Aggarwal, 2017).

#### 2.3.1 Outlier results

As with the machine learning method, the outlier method results varied by commodity. Outlier cutoffs for meat overall were an average outbreak size >60.60 individuals and a hospitalization to illness ratio of greater than 0.30; for beef and turkey, any serotype with an average outbreak size and hospitalization to illness ratio >0 were SoC; for chicken, an average outbreak size >14.50 and a ratio of >0; for pork, an average outbreak size >24.50 and a ratio >0.15 were SoC (Supplementary Table 2, Supplementary Figure 2). Serotypes identified as SoC for the four commodities and meat overall are presented in Table 1 and Figure 1.

### 2.4 Generating the serotypes of concern list

To generate our final list of SoC, any serotype that was identified by either the machine learning approach or the outlier approach was classified as a SoC (Table 1, Figure 1). Additionally, serotypes are marked as SoC for a given commodity if either approach identified it as such for that commodity. Combining the results of both methods results in a more holistic list, in alignment with our goals.

## 3 Obstacles and opportunities

### 3.1 Data limitations

Perhaps the most important limitation of our work is that NORS is a dynamic reporting system (reports can be modified, added, or removed at any time), so that future analyses may differ from ours due to the dynamic nature of the database. There are also limitations in the data available at the time of analysis. Outbreaks make up on average 10% of documented illnesses (Scallan et al., 2011; The Interagency Food Safety Analytics Collaboration, 2020), as such, this analysis does not take illnesses attributed to sporadic incidence into account, unlike the Jones study (Jones et al., 2008). Additionally, NORS is a voluntary reporting system and so does not represent all outbreaks in the U.S. Breaking down the data by specific source attribution commodities resulted in low sample sizes for some analyses. Beef and turkey for example, had few outbreak data (n_outbreaks_ = 37 and 31, respectively) and accordingly, more serotypes were identified for beef and turkey than chicken (n_outbreaks_ = 96) and pork (n_outbreaks_ = 63). If these data were parsed at the level of detail required for a business entity wanting to take actions in a specific production system, there are even less data available for analysis. Epidemiological data are naturally imperfect in that they contain information not just about the pathogen, but about the host and the environment as well. For example, Montevideo was identified as a beef SoC, yet the two largest beef-attributed Montevideo outbreaks can be traced back to a single caterer with a backyard chicken flock which may have contributed to the contamination of the beef prepared by the caterer (North Dakota Department of Health - Division of Disease Control, 2009). This final issue is challenging to tackle without parsing all individual outbreak reports, and there is not information at this level of detail for every outbreak. Researchers must be careful to be clear about the limits of their data as they identify SoC.

### 3.2 Machine learning is not a panacea

As expected, this case study did reveal candidate SoC that may have been “hidden” by traditional approaches. Many machine learning methods are rejected as “black boxes.” That is, the methods are poorly documented to a degree that no critical analysis can be performed. This can be remedied by clearly and accurately explaining the techniques used that resulted in the final output. Machine learning tools should not be treated as a black box, rather, we must focus on replicability and clear communication of research.

### 3.3 The outlier approach requires many decisions

Unlike the machine learning approach, the outlier approach required two major decisions to be made: what variables to use and how to define an outlier. While these decisions were made using expert knowledge, we have limited tools to quantitatively validate them. This may be the greatest weakness of the outlier approach, but it is also its strength as we can seamlessly incorporate expert knowledge into our methods.

### 3.4 Other considerations

In addition to the above obstacles, the issues of what data to include and the role of pathogen evolution must be considered. Incidence alone is not enough for the determination of SoCs because most cases of salmonellosis are self-limiting with at-home care, and to improve human health we need to target the most “dangerous” types of *Salmonella*. Similarly, including death in the SoC definition may also bias results because salmonellosis is usually not the only factor contributing to a death outcome—comorbidities are often present (Cummings et al., 2010). Therefore, we instead propose the inclusion of a measurement of disease severity (i.e., hospitalization to infection ratio) or another metric of pathogenicity other than death in SoC definitions. Furthermore, while this list of SoC represents a snapshot of the current *Salmonella* epidemiological landscape, pathogens are constantly evolving. For example, the Infantis strain containing the pESI plasmid has become a highly successful strain that now dominates the *Salmonella* isolated from poultry at harvest in the U.S. (McMillan et al., 2022). In the earlier 20^th^ century, successful control of serotype Gallinarum likely led to the emergence of Enteritidis as a dominant serotype in poultry (Rabsch et al., 2000). When targeting serotypes, we must be cautious to include evolutionary models to understand how best to manage them. Increased surveillance and re-visiting SoC definitions periodically will be required to help us stay ahead of *Salmonella* evolution. Finally, while serotyping has historically been instrumental in subtyping and understanding the diversity of *Salmonella*, evidence suggests that there are important differences even within serotypes (Cohn et al., 2021; Chen et al., 2022). As we move away from serotyping as the dominant subtyping method and toward genetics-based subtyping, ensuring “backwards compatibility” of new methods against SoC is key.

## 4 Conclusions

Defining a list of *Salmonella* serotypes to target to improve public health outcomes is a challenging yet critical task. Current approaches may oversimplify the true complexity of the *Salmonella* problem, leaving us to target only the most common serotypes. Yet, little evidence exists to suggest that control of the premier serotypes, such as Enteritidis or Typhimurium, will achieve the goal of decreasing *Salmonella* infection in humans. We developed frameworks for a quantitative, epidemiological method to define target serotypes for management and control and have produced a list of serotypes of concern (SoC) for the meat and poultry industry. The serotypes identified can be utilized by industry to target specific *Salmonella* and improves upon existing Key Performance Indicators by being epidemiologically validated. The development of rapid testing technologies, which target a suite of *Salmonella* serotypes based on shared features, could use this list as validation to ensure the tool will bring about the desired human health improvements. Further, using the code generated during this study and the CDC National Outbreak Reporting system, researchers and industry alike can tailor this analysis to their specific needs or update the analysis with new data over time.

New approaches to defining SoC must consider the holistic scope of host-pathogen-environment interactions, evolution, and comorbidities in the host while remaining scientifically and statistically supported. By incorporating these factors into new definitions of target serotypes, we believe there is great opportunity for advancing the control of *Salmonella* for the betterment of public health.

## Data availability statement

Publicly available datasets were analyzed in this study. These data can be found here: Centers for Disease Control National Outbreak Reporting System and https://github.com/tatumskatz/serotypesOfConcern.

## Author contributions

TK: Data curation, Formal analysis, Investigation, Methodology, Software, Visualization, Writing – original draft, Writing – review & editing. DH: Conceptualization, Methodology, Validation, Writing – review & editing. JS: Conceptualization, Writing – review & editing. TW: Conceptualization, Supervision, Writing – review & editing.
