# Supplementary material for: Identifying a list of Salmonella serotypes of concern to target for reducing risk of salmonellosis
Source: Front Microbiol. 2024 Feb 12;15:1307563. doi: 10.3389/fmicb.2024.1307563 (PMC10894960; doi:10.3389/fmicb.2024.1307563)
Supplement: Supplementary file 1 [file Data_Sheet_1.docx]

Supplementary Material

# Methods

## Statistical Software

All the following analyses were performed in the R statistical software computing environment (R Development Core Team, 2013) version 4.2.1 “Funny-Looking Kid” and using the RStudio integrated development environment (RStudio Team, 2020) “Spotted Wakerobin” release (2022.07.2 build 576) for macOS. All code generated for data cleaning, analyses, and the creation of Table 1 is available at <https://github.com/tatumskatz/serotypesOfConcern>.

## Epidemiological Data

Data were downloaded from Center for Disease Control’s National Outbreak Reporting System (NORS) on 1/18/2023 using the following methodology: mode of transmission = food; outbreak report has been finalized; onset year between 1999 and 2021; number of estimated primary illnesses greater than 1; genus = *Salmonella*; outbreaks with a confirmed or suspected etiology of *Salmonella*, including those with additional non-*Salmonella* etiologies reported. The data cleaning process occurred in five steps (Supplementary Figure 1). For analysis, data were truncated at 2009 due to differences in data collection methods and data management processes (Centers for Disease Control and Prevention, 2023). Outbreaks with multiple pathogen etiologies (including multiple *Salmonella* serotypes), unconfirmed etiologies, or without a confirmed serotype were removed from the dataset to avoid making assumptions about which pathogen or serotype was the actual cause of illness. Finally, outbreaks without an attributed food category were removed. Following data cleaning, 694 out of 3042 outbreaks remained for analysis. Outbreaks were subsequently split into “non-meat” and “meat” attributed sources, where “non-meat” included produce, plant products, aquatic animals (including fish and shellfish), game meat, fungi, eggs, dairy, and undefined meat products (“meat”, “other meat”, “poultry”, “other poultry”, and “meat-poultry”, n = 11). A source was only defined as “meat” if it was specifically identified as beef, chicken, pork, or turkey by its IFSAC level 4 category (Richardson et al., 2017). The “meat overall” group consisted of all beef, chicken, pork, and turkey-attributed outbreaks combined (Supplementary Figure 1).

## Machine Learning Approach

From the CDC NORS dataset, we calculated the number of outbreaks, illnesses, and hospitalizations for each serotype-year combination attributed to five sources: beef, chicken, pork, turkey, and all four of the previous categories combined (meat overall). To determine the optimal clustering method, an agglomerative nesting hierarchical cluster analysis (AGNES, (Kaufman and Rousseeuw, 1990; Maechler et al., 2022) was generated for each of the five sources using each of the following methods: unweighted pair-group arithmetic average (“average”), single linkage (“single”), complete linkage (“complete”), and Ward’s method (“ward”) (Ward, 1963; Maechler et al., 2022). The method which produced the highest agglomerative coefficient, which measures the clustering structure of the dataset, was used for further analysis (Supplementary Table 1). To determine the optimal number of clusters, elbow plots showing the within cluster sums of squares and average silhouette values per number of clusters (K) were generated (Kaufman and Rousseeuw, 1990) using the R package "factoextra" (Kassambara and Mundt, 2020). Plots which visualized cluster groupings were created for the optimal K which minimized the within cluster sums of squares and maximized the average silhouette values, as well as for K+1 using R package “factoextra” (Kassambara and Mundt, 2020, Supplementary Figures 3 through 7) The number of clusters was identified as a trade-off between the best K identified by the elbow plots while ensuring good visual separation. Outgroups were identified by visual analysis and marked as SoC if that serotype was present in the outgroup for more than two years, to eliminate singleton outbreaks. Finally, to recover the decision rules for how serotype-years were grouped by AGNES, a classification decision tree (Breiman et al., 1984) was trained on the group assignments using the R packages “rpart” (Therneau et al., 2022) and “rpart.plot”, and the decision rules are provided in Supplementary Table 1.

## Outlier Approach

From the CDC NORS dataset, the average outbreak size and hospitalization:illness ratio was calculated for each serotype. To define outliers, only serotypes which had average outbreak sizes and hospitalization to illness ratios greater than the third quartile were considered. Within that group, the difference between each serotype’s value and the 3^rd^ quartile divided by the interquartile range (IQR) was calculated. Any serotype whose score was greater than 1 (i.e., their distance from the 3^rd^ quartile was larger than the IQR) for both outbreak size and hospitalization:illness ratio was considered an outlier and therefore, a SoC. This analysis was repeated for five categories: beef, chicken, pork, turkey, and all four of the previous categories combined (meat overall) to generate five lists of outliers for their respective commodities (Supplementary Table 2, Supplementary Figure 2). Visualizations were developed using the R package “ggplot2” (Wickham 2016).

# Supplementary Figures


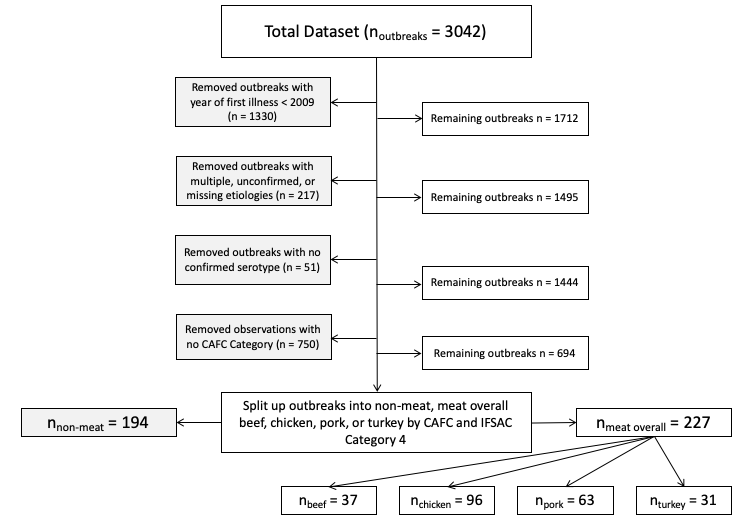


**Supplementary Figure 1.** Flowchart describing the data cleaning and sorting process performed on the CDC NORS dataset.

**Supplementary Table 1. Machine Learning Approach Results.** The table below provides results of the AGNES performed on each commodity group. The clustering method which produced the highest agglomerative coefficient is denoted with an asterisk. Best K (WSS) and Best K (Silhouette) provide the recommended number of clusters (K) to use based on minimizing the within-sum of squares and maximizing the average silhouette width. Chosen K and outlier groups were determined by the Best K recommendations and visual analysis of Supplementary Figures 2 through 6.

|  | Meat Overall | Beef | Chicken | Pork | Turkey |
| --- | --- | --- | --- | --- | --- |
| Agglomerative Coefficients | average: 0.977  single: 0.973  complete: 0.979  ward*: 0.990 | average: 0.990  single: 0.990  complete: 0.991  ward*: 0.992 | average: 0.987  single: 0.987  complete: 0.987  ward*: 0.989 | average: 0.988  single: 0.976  complete: 0.990  ward*: 0.994 | average: 0.988  single: 0.986  complete: 0.988  ward*: 0.994 |
| Best K (WSS) | 3 | 3 | 3 | 3 | 4 |
| Best K (Silhouette) | 2 | 2 | 2 | 2 | 3 |
| Chosen K and Groups | K=3; clusters 2 and 3 | K=3; clusters 2 and 3 | K=3; clusters 2 and 3 | K=4; clusters 3 and 4 | K=3; clusters 2 and 3 |
| Decision Rule | more than one outbreak per year | more than one outbreak per year | more than one outbreak per year | more than one outbreak per year, or one outbreak per year with at least 7 hospitalizations | more than one outbreak per year |
| Serotypes of Concern | Enteritidis, Heidelberg, I,4,[5],12:i:-, Infantis, Javiana, Montevideo, Newport, Typhimurium | Dublin, Enteritidis, Newport, Typhimurium, Uganda | Enteritidis | Enteritidis, I,4,[5],12:i:-, Typhimurium | Enteritidis, Hadar, Heidelberg, I,4,[5],12:i:-, Reading, Saintpaul, Typhimurium |

**Supplementary Table 2. Outlier Approach Results.** Table provides the calculate outlier cutoffs for the variables for each commodity. Serotypes with values greater than those cutoffs were labeled as serotypes of concern.

|  | Meat Overall | Beef | Chicken | Pork | Turkey |
| --- | --- | --- | --- | --- | --- |
| \|  \| \| --- \|   Hospitalizations to Illnesses Ratio | 0.30 | 0.00 | 0.00 | 0.15 | 0.00 |
| Average Number of Outbreaks | 60.60 | 0.00 | 14.50 | 24.50 | 0.00 |
| Serotypes of Concern | Reading | Braenderup, Dublin, Enteritidis, Heidelberg, I,4,[5],12:i:-, Infantis, Montevideo, Muenchen, Newport, Oranienburg, Potsdam, Typhimurium, Uganda | Blockley, Braenderup, Carmel, Heidelberg, I,4,[5],12:i:-, Infantis, Javiana, Montevideo, Saintpaul, Thompson, Typhimurium | Berta, I,4,[5],12:i:-, Infantis, Schwarzengrund | Anatum, Berta, Enteritidis, Hadar, Heidelberg, Javiana, Muenchen, Newport, Reading, Saintpaul, Schwarzengrund, Typhimurium |


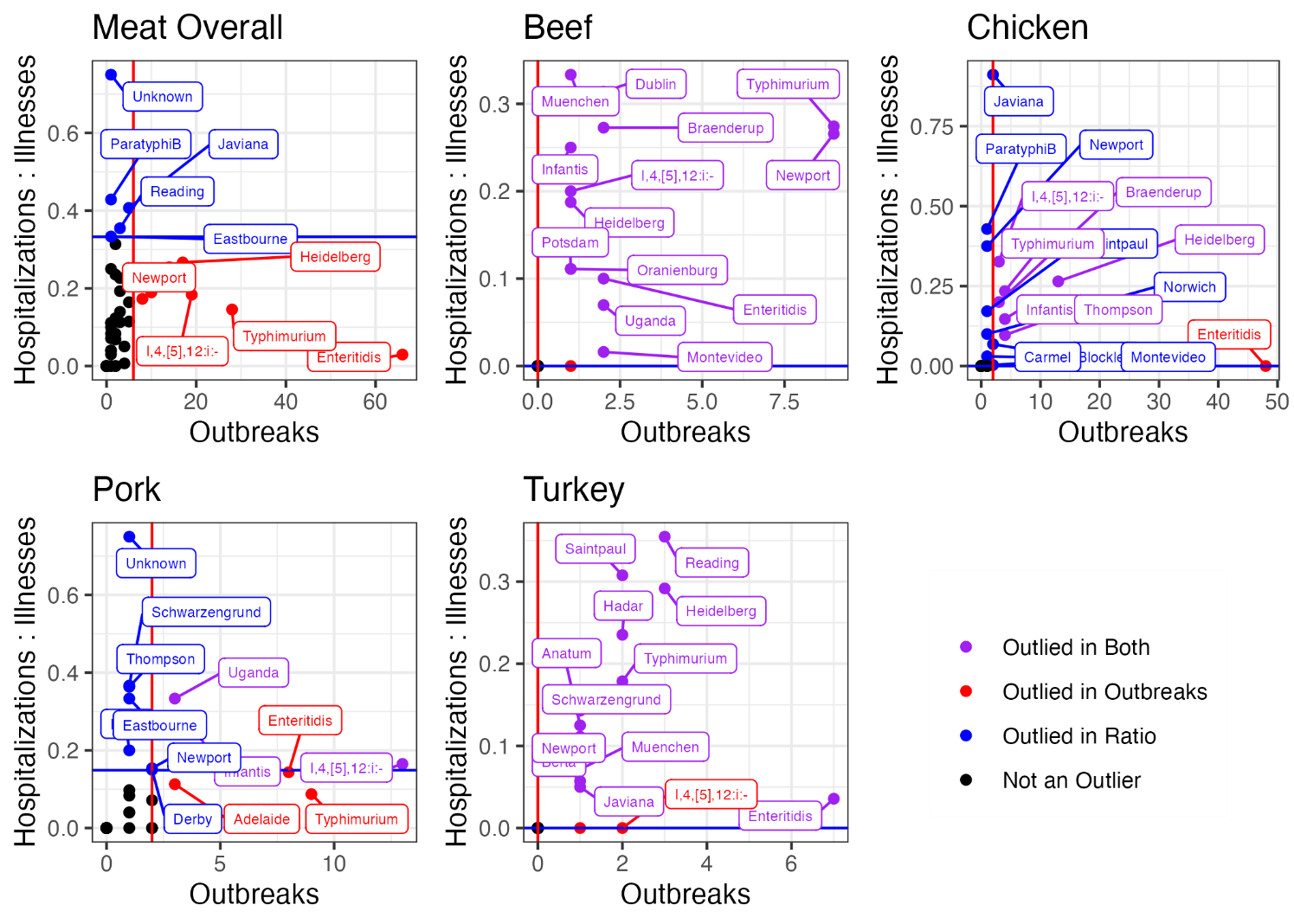


**Supplementary Figure 2. Results of the Outlier Approach.** Outlier cutoffs for the ratio of hospitalizations to illnesses (blue line) and the average number of outbreaks (red line) were calculated for each commodity. Any serotype which fell outside of either of those cutoffs is labeled on the plot; serotype names appear in blue if they are outlied in their hospitalizations to illnesses ratio, red if they are outlied in their average number of outbreaks, and in purple if they are outlied in both variables.


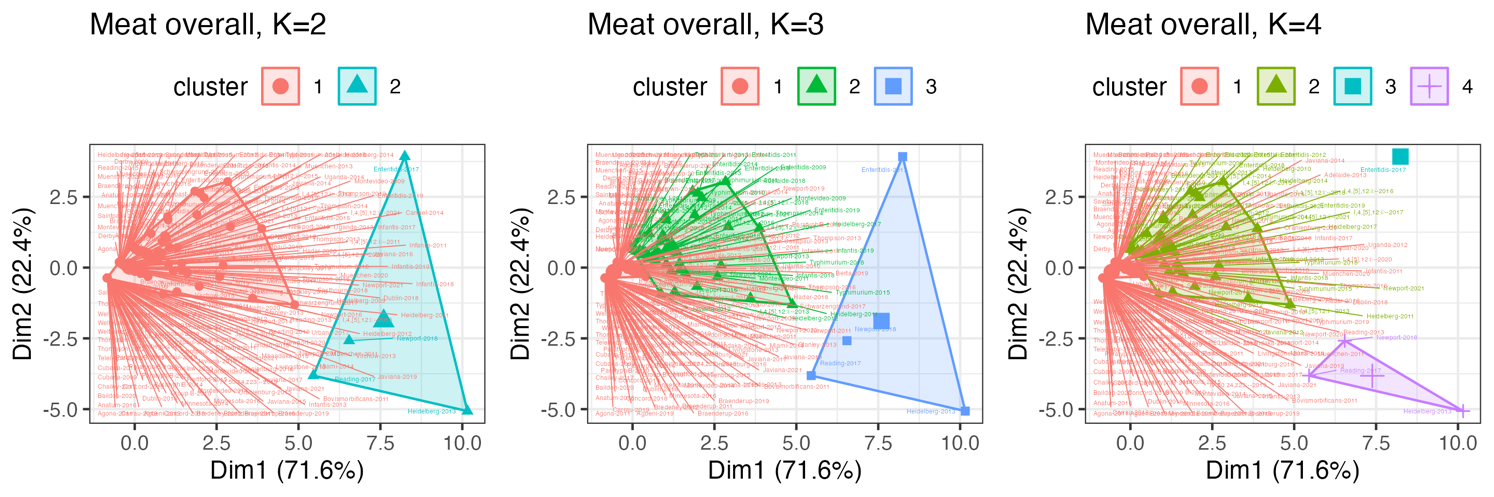


**Supplementary Figure 3. Visualization of cluster groupings for K=2:4, for meat overall.** K=3 was selected as the best K and serotypes within clusters 2 and 3 were marked as serotypes of concern if they appeared in those clusters for at least two years.


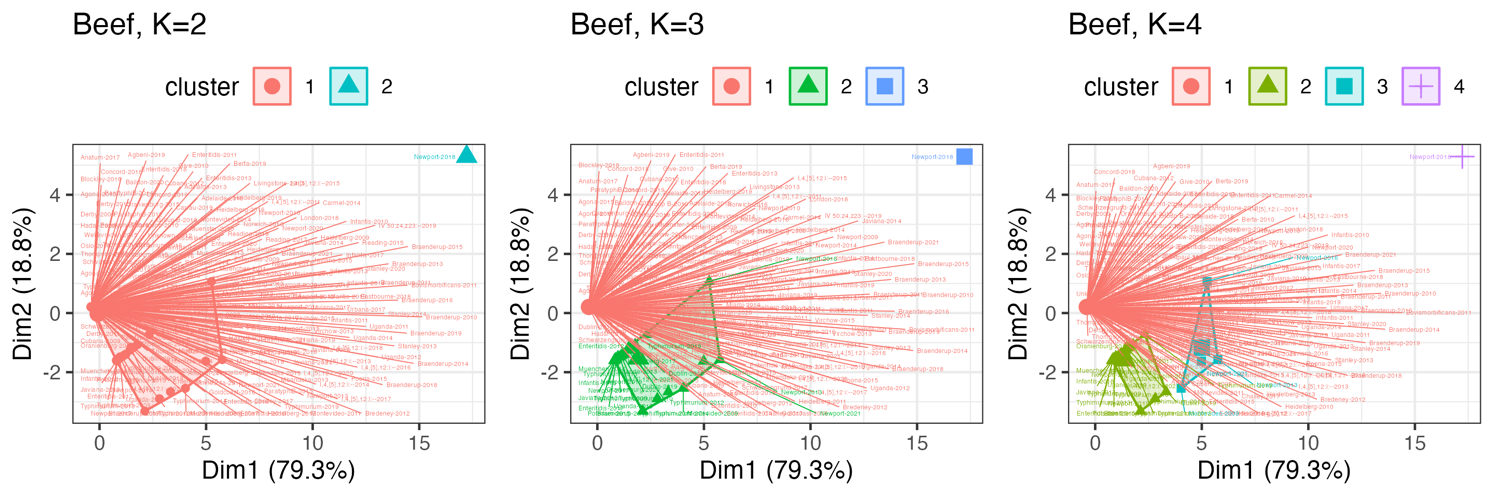


**Supplementary Figure 4. Visualization of cluster groupings for K=2:4, for beef.** K=3 was selected as the best K and serotypes within clusters 2 and 3 were marked as serotypes of concern if they appeared in those clusters for at least two years.


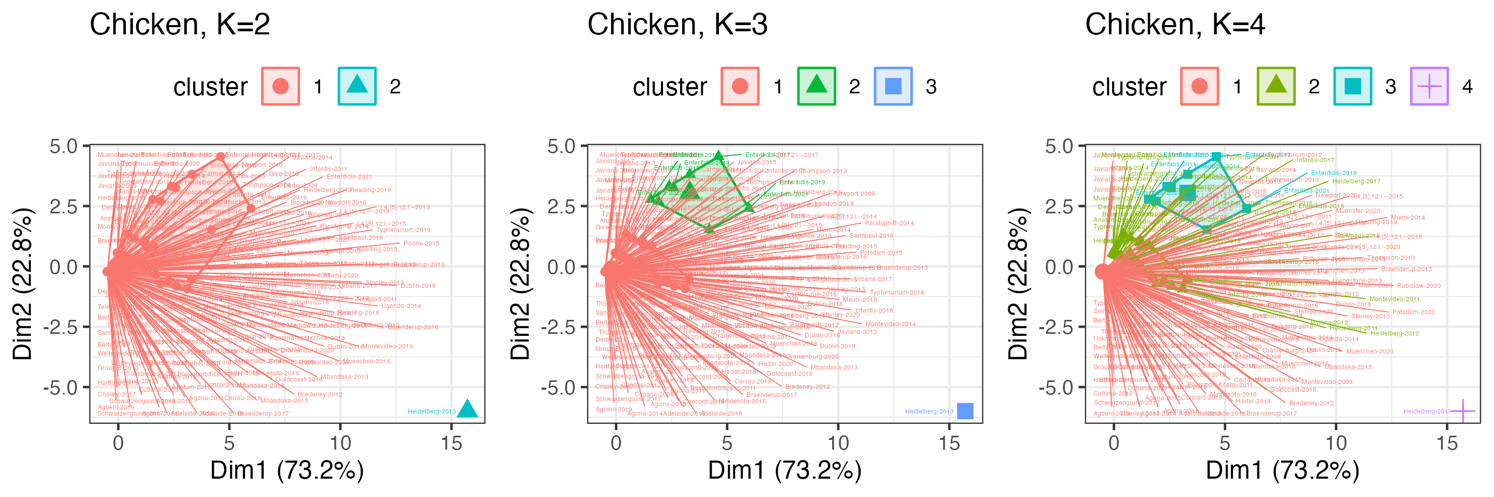


**Supplementary Figure 5. Visualization of cluster groupings for K=2:4, for chicken.** K=3 was selected as the best K and serotypes within clusters 2 and 3 were marked as serotypes of concern if they appeared in those clusters for at least two years.


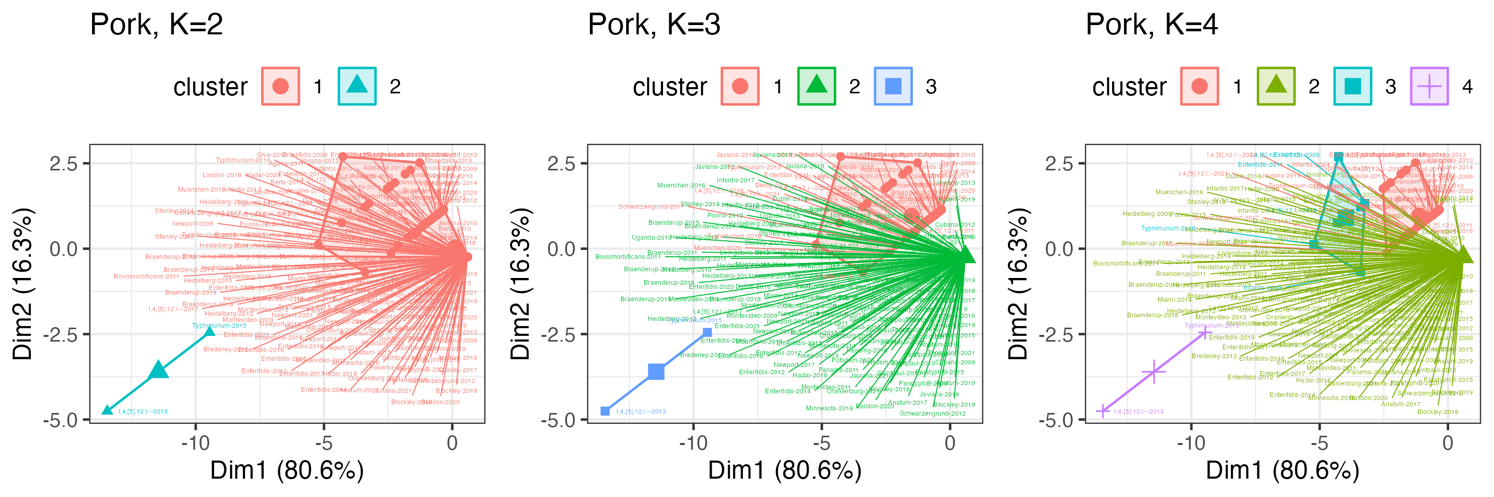


**Supplementary Figure 6. Visualization of cluster groupings for K=2:4, for pork.** K=4 was selected as the best K and serotypes within clusters 3 and 4 were marked as serotypes of concern if they appeared in those clusters for at least two years.


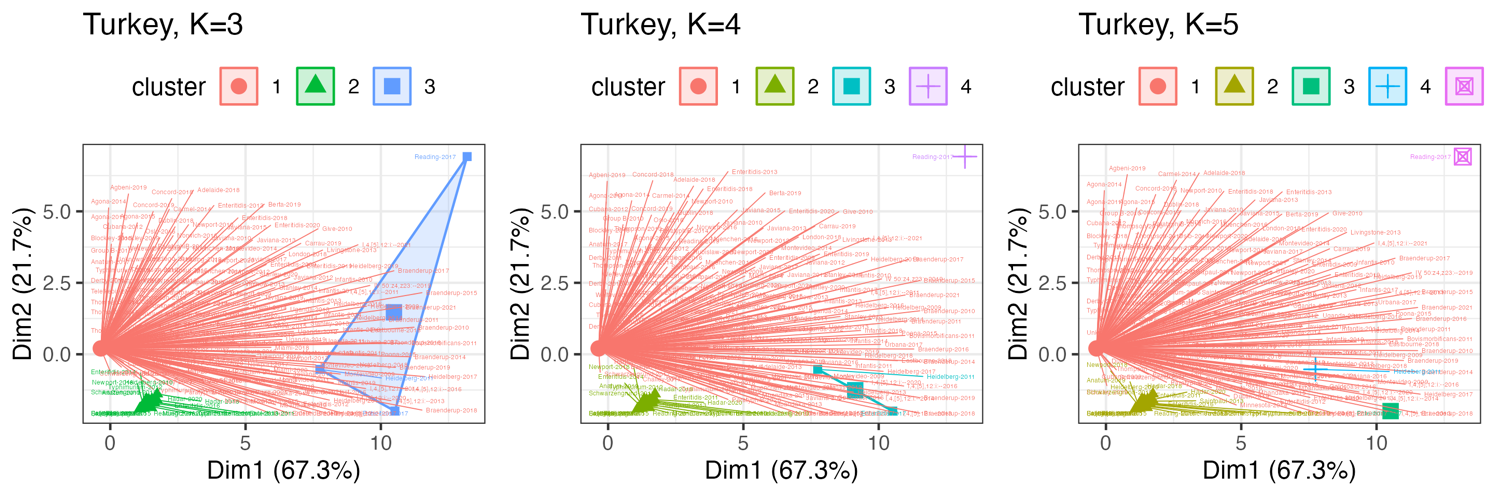


**Supplementary Figure 7. Visualization of cluster groupings for K=3:5, for turkey.** K=3 was selected as the best K and serotypes within clusters 2 and 3 were marked as serotypes of concern if they appeared in those clusters for at least two years.

# References

Breiman, L., Friedman, J. H., Olshen, R. A., and Stone, C. J. (1984). *Classification And Regression Trees*. 1st ed. Routledge doi: 10.1201/9781315139470.

Centers for Disease Control and Prevention (2023). National Outbreak Reporting System.

Kassambara, A., and Mundt, F. (2020). Package “factoextra” Type Package Title Extract and Visualize the Results of Multivariate Data Analyses. Available at: https://github.com/kassambara/factoextra/issues.

Kaufman, L., and Rousseeuw, P. J. (1990). *Finding Groups in Data: An Introduction to Cluster Analysis*. Wiley.

Maechler, M., Rousseeuw, P., Struyf, A., Hubert, M., Hornik, K., Studer, M., et al. (2022). Package “cluster.” Available at: https://orcid.org/0000-0001-9143-4880.

Milborrow, S. (2022). rpart.plot: Plot 'rpart' Models: An Enhanced Version of 'plot.rpart'. R package version 3.1.1, Available at: https://CRAN.R-project.org/package=rpart.plot.

R Development Core Team (2013). R: a language and environment for statistical computing.

Richardson, L. C., Bazaco, M. C., Parker, C. C., Dewey-Mattia, D., Golden, N., Jones, K., et al. (2017). An Updated Scheme for Categorizing Foods Implicated in Foodborne Disease Outbreaks: A Tri-Agency Collaboration. *Foodborne Pathog. Dis.* 14, 701–710. doi: 10.1089/fpd.2017.2324.

RStudio Team (2020). RStudio: Integrated Development for R. Available at: www.rstudio.com.

Therneau, T., Atkinson, B., and Ripley, B. (2022). Package “rpart.” Available at: https://cran.r-project.org/package=rpart.

Ward, J. H. (1963). Hierarchical Grouping to Optimize an Objective Function.

Wickham, H. (2016). ggplot2: Elegant Graphics for Data Analysis. Springer-Verlag New York. Available at: https://ggplot2.tidyverse.org.
